# The Functional Side of Multiple Endocrine Neoplasia Type 1-Associated Adrenal Disease: Mild Autonomous Cortisol Secretion

**DOI:** 10.3390/diseases14090322

**Published:** 2026-09-05

**Authors:** Roberta Modica, Michele Coletta, Elio Benevento, Alessia Liccardi, Roberto Minotta, Gianfranco Di Iasi, Massimo Di Nola, Roberta Pia Bertenni, Annamaria Colao

**Affiliations:** 1 Endocrinology, Diabetology and Andrology Unit, Department of Clinical Medicine and Surgery, University of Naples Federico II, 80131 Naples, Italy; michele99coletta@gmail.com (M.C.); alessia.liccardi@yahoo.com (A.L.); robertominotta@gmail.com (R.M.); gianfrancodiiasi@gmail.com (G.D.I.); massimodinola96@gmail.com (M.D.N.); bertennirobertapia@gmail.com (R.P.B.); colao@unina.it (A.C.); 2 UNESCO Chair, Education for Health and Sustainable Development, University of Naples Federico II, 80131 Naples, Italy

**Keywords:** multiple neuroendocrine neoplasia type 1, adrenal lesion, mild autonomous cortisol secretion, metabolic syndrome

## Abstract

**Background/Objectives**: Multiple endocrine neoplasia type 1 (MEN1) is a rare hereditary syndrome characterized by primary hyperparathyroidism, duodeno-pancreatic and pituitary neuroendocrine tumors. Adrenal lesions are acknowledged manifestations of MEN1, but their functional characterization remains limited. Mild autonomous cortisol secretion (MACS) is associated with cardiometabolic risk and skeletal involvement in sporadic adrenal incidentaloma, significantly impacting patient morbidity, but data in MEN1 are lacking. The aims of the study were to estimate the prevalence of MACS in adult patients with MEN1 and radiological evidence of adrenal involvement, and to evaluate the associated biochemical, cardiometabolic, and skeletal features. **Methods:** This retrospective single-center observational study included adult patients with clinical, familial, or genetic MEN1 and adrenal involvement. MACS was defined as serum cortisol >1.8 µg/dL after a 1 mg overnight dexamethasone suppression test in the absence of overt Cushing syndrome. Cardiometabolic and skeletal characteristics were compared according to MACS status. **Results:** Among 101 MEN1 patients, 38 had adrenal involvement and 22 underwent complete hormonal evaluation. MACS was identified in 15 of the 22 patients (68.2%). Patients with MACS had significantly lower baseline ACTH concentrations and showed a trend toward a higher prevalence of metabolic syndrome. No significant differences were observed in osteoporosis or fracture prevalence. **Conclusions:** This is the first study specifically evaluating the prevalence of MACS in MEN1 patients with adrenal lesions. MACS appears to be more common than in sporadic adrenal incidentalomas and may represent an important factor for improving clinical characterization and tailoring patient management. Larger prospective studies are needed to define the optimal follow-up strategy.

## 1. Introduction

Multiple endocrine neoplasia type 1 (MEN1) is a rare autosomal dominant inherited syndrome, with an estimated prevalence of 3–20 per 100,000 individuals and high age-related penetrance, with a slight female preponderance [1,2]. It is mainly characterized by primary hyperparathyroidism (pHPT), duodenopancreatic neuroendocrine tumors (dpNET), and pituitary neuroendocrine tumors (PitNET) [3,4]. Adrenal lesions including unilateral or bilateral adrenocortical adenomas (AA) and diffuse adrenal hyperplasia (AH) are increasingly recognized in MEN1, often during abdominal imaging performed for disease surveillance [5,6]. AA occur in around 40% of patients by age 80 years, although estimated prevalence varies substantially across cohorts [5]. Importantly, the functional characterization of MEN1-associated adrenal lesions remains poorly defined, with most reports focusing on prevalence, morphology, growth and radiological follow-up [5,6,7]. Adrenal lesions are frequently encountered outside the context of hereditary syndromes, with autopsy studies suggesting an overall prevalence of approximately 2%; adrenal incidentaloma (AI) prevalence increases with age, being around 3% in adults older than 50 years and up to 10% in those older than 80 years [8]. Mild autonomous cortisol secretion (MACS) refers to ACTH-independent cortisol hypersecretion in patients with AA or AH without the clinical features of overt Cushing syndrome, such as proximal muscle weakness, skin fragility, or purple striae. Biochemically, MACS is currently defined by a post–1 mg overnight dexamethasone suppression test (1 mg-DST) serum cortisol concentration >1.8 μg/dL (50 nmol/L), indicating insufficient cortisol suppression [8,9]. Thus, the diagnosis of MACS requires the integration of biochemical, clinical, and radiological finds [8,9]. Dehydroepiandrosterone sulfate (DHEA-S) may provide complementary information as a potential additional marker of chronic ACTH suppression in MACS [10,11]. Among AI, MACS accounts for approximately 20–50% of cases, although reported prevalence varies widely owing to differences in study design, patient selection, and diagnostic definitions of cortisol excess [8]. Importantly MACS has been associated with cardiometabolic comorbidities, including hypertension, diabetes mellitus, and dyslipidemia in sporadic AI cohorts [8,12]. Whether MACS and related comorbidities are identified in MEN1 patients is largely unknown, also due to the complexity of the syndrome with different disease-specific risks [5,6,8,13]. This is a relevant knowledge gap, as MEN1 patients develop multiple endocrine neoplasms with significant disease-related mortality. Indeed, MEN1 patients have lower life expectancy than general population, with dpNET representing the most common cause of death [14]. Cardiometabolic morbidities and mortality appear increased in patients with MACS and AI, compared with patients with non-functioning incidentalomas, despite strong conclusions on causality remain debated. In MEN1, MACS may represent an additional and potentially modifiable contributor to mortality risk, providing a strong rationale for investigating its prevalence and clinical significance in MEN1. The clinical interpretation of MACS in MEN1 may be hampered by disease-specific manifestations and treatments. Therefore, MACS, DHEA-S suppression, cardiometabolic disease, and bone health should be interpreted cautiously in MEN1 cohorts, considering the complex endocrine background that may impact on clinical features. The coexistence of multiple endocrine disorders affecting metabolism, bone and cardiometabolic health makes MEN1 substantially different from sporadic AI cohorts, reinforcing the need for disease-specific studies rather than extrapolation from the general population. The primary aim of this study was to determine the prevalence of MACS in a cohort of adult MEN1 patients and radiological evidence of adrenal lesions (AA or AH). Secondary aims were to characterize the biochemical and clinical phenotype associated with MACS by evaluating also serum DHEA-S concentrations; describing cardiometabolic comorbidities and bone health according to MACS status; and exploring the association between baseline DHEA-S concentrations and lack of cortisol suppression after 1 mg-DST.

## 2. Materials and Methods

### 2.1. Study Population

The study was designed as a retrospective, single-center, observational cohort study based on cross-sectional analysis of clinical, biochemical, and radiological data. Patients were followed at the ENETS Center of Excellence of the Endocrinology, Diabetology and Andrology Unit, University of Naples Federico II, Naples, Italy. The study included patients with MEN1 syndrome followed at the center between January 2006 and June 2026. 

### 2.2. Inclusion and Exclusion Criteria

Eligible patients were adults with clinical, familial, or genetic MEN1 diagnosis and radiological evidence of AA or unilateral or bilateral diffuse adrenal enlargement consistent with micro- or macronodular AH. Clinical MEN1 was defined by the presence of at least two major MEN1-associated endocrine tumors; familial MEN1 by the occurrence of at least one MEN1-associated tumor and a first-degree relative with genetically confirmed MEN1; and genetic MEN1 by the identification of a germline MEN1 pathogenic variant. Radiological evidence of AA was defined by the presence of adrenal lesion with benign imaging features consistent with a benign adenoma, including an unenhanced attenuation value ≤10 Hounsfield units on non-contrast CT. Exclusion criteria included overt Cushing syndrome, pheochromocytoma or other functioning adrenal lesions, adrenocortical carcinoma, insufficient clinical, biochemical, or radiological data for the planned analyses, and treatment with medications known to interfere with the interpretation of the 1 mg-DST or cortisol measurements. 

### 2.3. Data Collection

Clinical, biochemical, and radiological data were retrospectively collected from available medical records, laboratory and imaging reports. Collected data included demographic characteristics (age and sex), anthropometric measures, MEN1-related clinical manifestations, adrenal imaging features, chronic medications, cardiometabolic comorbidities including hypertension, impaired glucose and lipid metabolism, overweight or obesity, bone health evaluation including bone mineral density (BMD) and fragility fractures. 

### 2.4. Hormonal and Biochemical Assessment

Baseline biochemical–hormonal profiling included morning assessment of ACTH, cortisol, and DHEA-S at 08:00 AM, 24 h urinary free cortisol (24h-UFC). All hormonal determinations were performed in accredited laboratories affiliated with the Italian National Health Service, using chemiluminescent immunoassay. The methodology used for each analyte was the same throughout the study period, ensuring consistency and comparability of results. The overnight 1 mg-DST was performed on the day following baseline assessment. Patients were instructed to take 1 mg of oral dexamethasone at 11:00 PM; plasma ACTH and serum cortisol were collected at 08:00 AM the following morning. MACS was defined as serum cortisol concentration >1.8 µg/dL following 1 mg-DST, equivalent to >50 nmol/L, in the absence of clinical signs and symptoms of overt Cushing syndrome. Patients with post 1 mg-DST cortisol ≤1.8 µg/dL were classified as not having MACS (non-MACS). DHEA-S suppression was defined as a baseline DHEA-S concentration below the lower limit of the age- and sex-specific reference interval. Anthropometric parameters included weight, height, body mass index (BMI), calculated as weight (kg) divided by height squared (m^2^) and waist circumference. Cardiometabolic variables included arterial hypertension, disorder of glucose and lipid metabolism, overweight or obesity, and metabolic syndrome defined by the presence of at least three of the following American Heart Association/National Heart, Lung and Blood Institute (AHA/NHLBI) criteria: waist circumference ≥88 cm in women or ≥102 cm in men,; HDL <50 mg/dL in women or <40 mg/dL in men; triglycerides ≥150 mg/dL; blood pressure ≥130/85 mmHg, and/or use of antihypertensive medications; and fasting blood glucose ≥100 mg/dL or glucose-lowering treatment [15]. Arterial hypertension was defined as a documented diagnosis, ongoing antihypertensive treatment, or the average of office blood pressure measurements meeting the diagnostic thresholds for hypertension with stage 1 hypertension defined as systolic blood pressure of 130–139 mmHg or diastolic blood pressure of 80–89 mmHg, and stage 2 hypertension as systolic blood pressure ≥140 mmHg or diastolic blood pressure ≥ 90 mmHg according to guidelines [16]. Glucose variables included fasting glucose, glycated hemoglobin (HbA1c), diagnosis of diabetes mellitus or impaired fasting glucose according to guidelines and glucose-lowering medications [17]. Lipid variables included total cholesterol, HDL cholesterol and triglycerides and the use of lipid-lowering medications. Lipid metabolism disorders included a documented diagnosis of dyslipidemia, ongoing lipid-lowering treatment, or lipid values above risk-adapted guideline goals [18].

### 2.5. Anthropometrics Data

Overweight and obesity were classified according to BMI (overweight 25–29.9 kg/m^2^, obesity BMI ≥ 30.0 kg/m^2^). Obesity was further classified as class I (BMI 30–34.9 kg/m^2^), class II (BMI 35–39.9 kg/m^2^), and class III (BMI ≥40 kg/m^2^), according to the European Association for the Study of Obesity (EASO) [19]. 

### 2.6. Bone Health Data

Bone health was assessed by BMD measured using dual-energy X-ray absorptiometry (DXA), diagnosis of osteopenia or osteoporosis, reduced BMD values interpreted according to sex and age, and radiological evidence of vertebral fractures evaluated by thoracolumbar spine radiography with vertebral morphometry and/or vertebral fracture assessment. In postmenopausal women and men aged ≥50 years, osteoporosis was defined by a T-score ≤−2.5 standard deviations (SD) at the femoral neck or lumbar spine. Low bone mass/osteopenia was defined by a T-score between −1.0 and −2.5 SD and was considered a densitometric category for descriptive purposes rather than a disease entity. In premenopausal women and men < 50 years, BMD was interpreted using Z-score, with values ≤ −2.0 SD defined as below the expected range for age. Vertebral fractures were identified by lateral thoracolumbar spine radiography using vertebral morphometry/vertebral fracture assessment when available and classified according to the semiquantitative Genant method [20].

### 2.7. Statistical Analysis

Continuous variables were reported as mean ± standard deviation and min–max range. Categorical variables were reported as absolute frequencies and percentages. The prevalence of MACS was reported as n/N, percentage, and exact 95% confidence interval. In addition to recorded categorical variables, selected cardiometabolic categories were derived from available data using predefined rules: overweight/obesity was defined as BMI ≥ 25 kg/m^2^, obesity as BMI ≥ 30 kg/m^2^, impaired fasting glucose as fasting glucose 100–125 mg/dL in the absence of diabetes medication, and dyslipidemia as lipid-lowering therapy, low HDL cholesterol (<40 mg/dL in men or <50 mg/dL in women), or triglycerides ≥150 mg/dL. Between-group comparisons for continuous variables were performed using the Mann–Whitney test because of the limited sample size and expected non-normal data distribution. Between-group comparisons for categorical variables were performed using Fisher exact test or Pearson chi-square test, as appropriate according to expected cell counts. The significance threshold was set at *p* < 0.05. Statistical analyses were performed using R version 4.6.0.

## 3. Results

### 3.1. Study Population

Among 101 MEN1 patients (*n* = 59, 58.4% women; *n* = 42, 41.6% men) retrospectively screened for adrenal lesions, 38 patients (37.6%, women *n* = 18, 47.4%; men *n* = 20, 52.6%) had radiological evidence of AA or AH. Sixteen patients were excluded due to insufficient clinical or biochemical data. The final cohort included 22 patients (22/38, 57.9%), adrenal lesions, and hormonal assessment including 1 mg-DS. All 22 patients included in the final analytic cohort underwent genetic testing and had a genetically confirmed MEN1 diagnosis; 15 (68.2%) were familial cases and 7 (31.8%) were index cases. MEN1-related manifestations included dpNET in all patients, pHPT in 20 (90.9%), and PitNET in 13 (59.1%). Among the 22 patients with dpNETs, 21 (95.5%) had non-functioning tumors and 1 (4.5%) had a gastrinoma. Among the 13 patients with PitNETs, five (38.5%) had macroadenomas and eight (61.5%) had microadenomas. Regarding functional status, 6/13 PitNETs (46.2%) were non-functioning, 6/13 (46.2%) were prolactin-secreting, and 1/13 (7.7%) was a mixed PRL/GH-secreting tumor. SSA therapy was recorded in 20 patients (90.9%). Adrenal involvement was bilateral in 10 patients (45.5%), left-sided in nine (40.9%), and right-sided in three (13.6%). Among the 22 patients, 15 (68.2%) had AA and seven (31.8%) had AH. MACS was identified in 15 patients, corresponding to a prevalence of 68.2% (exact 95% CI, 45.1–86.1%). Seven out of 22 patients (31.8%) did not meet the biochemical criterion for MACS. DHEA-S suppression was observed in 12 (54.5%) patients, whereas 10 (45.5%) showed no DHEA-S suppression. Overweight or obesity (BMI ≥ 25 kg/m^2^) was present in 16/22 (72.7%) patients. Among the 22 patients, 11 (50%) had arterial hypertension, and were receiving antihypertensive treatment. Impaired fasting glucose in the absence of glucose-lowering medication was present in 3/22 (13.6%) patients. Diabetes medication was recorded in 10/22 (45.5%) patients. Dyslipidemia defined according to available lipid data and/or lipid-lowering therapy was present in 14/22 (63.6%) patients. Lipid-lowering therapy was recorded in 12/22 (54.5%) patients. Metabolic syndrome was recorded in 5/22 (22.7%) patients. Bone health data were available in 21/22 (95.5%) patients:osteoporosis was recorded in 12 (57.1%) patients among whom fractures were radiologically detected in 7 (31,3%); osteoporosis medication was recorded in 7 (31.8%) patients. Detailed baseline patient characteristics and continuous variables for the overall study population are summarized in Table 1 and Table 2, respectively. 

### 3.2. Comparison Between Patients with and Without Mild Autonomous Cortisol Secretion

MACS and non-MACS groups did not differ significantly in age (55.8 ± 11.5 years [range, 36–75] vs. 60.6 ± 14.8 years [range, 41–80], respectively; *p* = 0.480). Sex distribution differed significantly between the MACS and non-MACS groups (overall *p* = 0.020). Men accounted for 11/15 patients (73.3%) in the MACS group and 1/7 patients (14.3%) in the non-MACS group, whereas women accounted for 4/15 (26.7%) and 6/7 patients (85.7%), respectively. Baseline ACTH differed significantly between MACS and non-MACS group: 17.8 ± 4.7 pg/mL [range, 10–28.2; *n* = 15] vs. 41.7 ± 26.4 pg/mL [range, 16–95.6; *n* = 7] (*p* = 0.008). No statistically significant differences were observed between MACS and non-MACS groups in morning cortisol levels or 24h-UFC: the morning cortisol was 16.0 ± 6.1 µg/dL [range, 8.2–30.3; *n* = 15] vs. 19.4 ± 8.2 µg/dL [range, 10.4–36.6; *n* = 7] (*p* = 0.259), and 24h-UFC was 212.4 ± 144 µg/24 h [range, 33–477; *n* = 14] vs. 169.6 ± 109 µg/24 h [range, 21–307; *n* = 7] respectively (*p* = 0.526). Post 1 mg-DST cortisol was significantly higher in MACS than in non-MACS group, as expected (2.5 ± 0.7 µg/dL [range, 1.8–3.9; *n* = 15] vs. 1.2 ± 0.3 µg/dL [range, 0.9–1.8; *n* = 7]; *p* < 0.001). Although DHEA-S concentrations did not differ significantly between MACS and non-MACS groups: 45 ± 58.1 µg/dL [range, 2–226.1; *n* = 15] vs. 96.3 ± 89.8 µg/dL [range, 11–232.6; *n* = 7], respectively (*p* = 0.139); DHEA-S suppression was observed in 10/15 (66.7%) and 2/7 (28.6%) patients, respectively (*p* = 0.172). Mean BMI did not differ significantly between MACS and non-MACS groups: 27.8 ± 2.7 kg/m^2^ [range, 22.6–31.6; *n* = 15] vs. 28.2 ± 9.5 kg/m^2^ [range, 21.7–48.8; *n* = 7] respectively (*p* = 0.147). Overweight or obesity (BMI ≥ 25 kg/m^2^) was present 12/15 (80%) patients in MACS and 4/7 (57.1%) patients in non-MACS group (*p* = 0.334), while obesity alone (BMI ≥30 kg/m^2^) was observed in 2/15 (13.3%) and 1/7 (14.3%) patients, respectively (*p* = 1.000). Arterial hypertension was present in 6/15 (40%) and 5/7 (71.4%) patients in MACS and non-MACS groups, respectively (*p* = 0.361). Impaired fasting glucose in the absence of glucose-lowering medication was present in 2/15 (13.3%) patients in MACS and 1/7 (14.3%) patients in non-MACS (*p* = 1.000). Diabetes medication was recorded in 10/22 (45.5%) patients, including 8/15 (53.3%) patients in MACS and 2/7 (28.6%) patients in non-MACS (*p* = 0.381). No statistically significant differences was observed in fasting glucose (117.1 ± 33.3 mg/dL [range, 86–219; *n* = 15] vs. 102.6 ± 21.3 mg/dL [range, 82–136; *n* = 7]; *p* = 0.307), or HbA1c (6.0 ± 1.1% [range, 4.3–9.1; *n* = 15] vs. 5.6 ± 0.9% [range, 4.3–7.1; *n* = 7] *p* = 0.502). Dyslipidemia defined according to available lipid data and/or lipid-lowering therapy was present in 10/15 (66.7%) patients in MACS and 4/7 (57.1%) patients in non-MACS group (*p* = 1.000). Lipid-lowering therapy was recorded in 9/15 (60%) patients in MACS and 3/7 (42.9%) patients in non-MACS group (*p* = 0.652). Although no statistically significant differences were observed between MACS and non-MACS group in total cholesterol (156.5 ± 39.1 mg/dL [range, 97–222; *n* = 15] vs. 162 ± 30.5 mg/dL [range, 114–197; n = 7]; *p* = 0.724), LDL cholesterol (85.7 ± 28.5 mg/dL [range, 47–129; *n* = 15] vs. 75.7 ± 18.9 mg/dL [range, 43–95; *n* = 7]; *p* = 0.378), triglycerides (96.7 ± 30.8 mg/dL [range, 32–138; *n* = 15] vs. 95.3 ± 28.9 mg/dL [range, 61–136; *n* = 7]; *p* = 0.944), HDL cholesterol was lower in MACS than in non-MACS group, with a trend toward statistical significance (53.2 ± 13.4 [range, 35–82; *n* = 15] vs. 68 ± 18.1 mg/dL [range, 42–100; *n* = 7]; *p* = 0.057). Metabolic syndrome was recorded in 4/15 (26.7%) patients in MACS and 1/7 (14.3%) patients in non-MACS group (*p* = 1.000). SSA therapy was recorded in 20/22 (90.9%) patients, including 13/15 (86.7%) patients in MACS and 7/7 (100%) patients in non-MACS (*p* = 1.000). Bone health data were available in 21/22 (95.5%) patients: lumbar T/Z-score was −0.7 ± 1.5 SD (range, −3.2 to 1.2; *n* = 14) in MACS and −1.4 ± 1.1 SD (range, −2.5 to 0.6; *n* = 7) in non-MACS (*p* = 0.247); femoral neck T/Z-score was −0.7 ± 1.5 SD (range, −2.5 to 2.9; *n* = 14) in MACS and −2.3 ± 1.1 SD (range, −3.3 to −0.5; *n* = 7) in non-MACS group (*p* = 0.023). Osteoporosis was recorded in 7/14 (50%) patients in MACS and 5/7 (71.4%) patients in non-MACS group (*p* = 0.642). Radiological or clinical fracture was recorded in 5/14 (35.7%) patients in MACS and 2/7 (28.6%) patients in non-MACS group (*p* = 1.000). Osteoporosis medication was recorded in 4/15 (26.7%) patients in MACS and 3/7 (42.9%) patients in non-MACS group (*p* = 0.630). Osteopenia was recorded in 5/14 (35.7%) patients in MACS and 1/7 (14.3%) patients in non-MACS group (*p* = 0.613). PHPT was recorded in 20/22 (90.9%) patients, including 13/15 (86.7%) patients in MACS and 7/7 (100%) patients in non-MACS (*p* = 1.000). PitNET was recorded in 8/15 (53.3%) patients in MACS group and 5/7 (71.4%) patients in non-MACS group. Among patients with PitNETs, macroadenomas were identified in 3/8 patients (37.5%) in MACS group and 2/5 patients (40.0%) in non-MACS group, whereas microadenomas were identified in 5/8 (62.5%) and 3/5 patients (60.0%), respectively. PitNET size distribution did not differ significantly between the two groups (*p* = 1.000). Furthermore, non-functioning tumors were recorded in 3/8 (37.5%) and 3/5 (60.0%), prolactin-secreting tumors in 4/8 (50.0%) and 2/5 (40.0%), and a mixed PRL/GH-secreting tumor in 1/8 (12.5%) and 0/5 (0%), in MACS and non-MACS groups, respectively. Overall, in this cohort of 22 patients with MEN1, AA or AH, and available 1 mg-DST data, MACS was frequent, being identified in approximately two-thirds of patients. DHEA-S suppression was more common among patients with MACS than among those without MACS, whereas cardiometabolic and skeletal comorbidities were common in both groups. Regarding bone health, femoral neck T/Z-score was significantly lower in the non-MACS group, while no other cardiometabolic or skeletal between-group differences reached statistical significance. Clinical and biochemical characteristics according to MACS status are presented in Table 3.

## 4. Discussion

**Figure 1:**
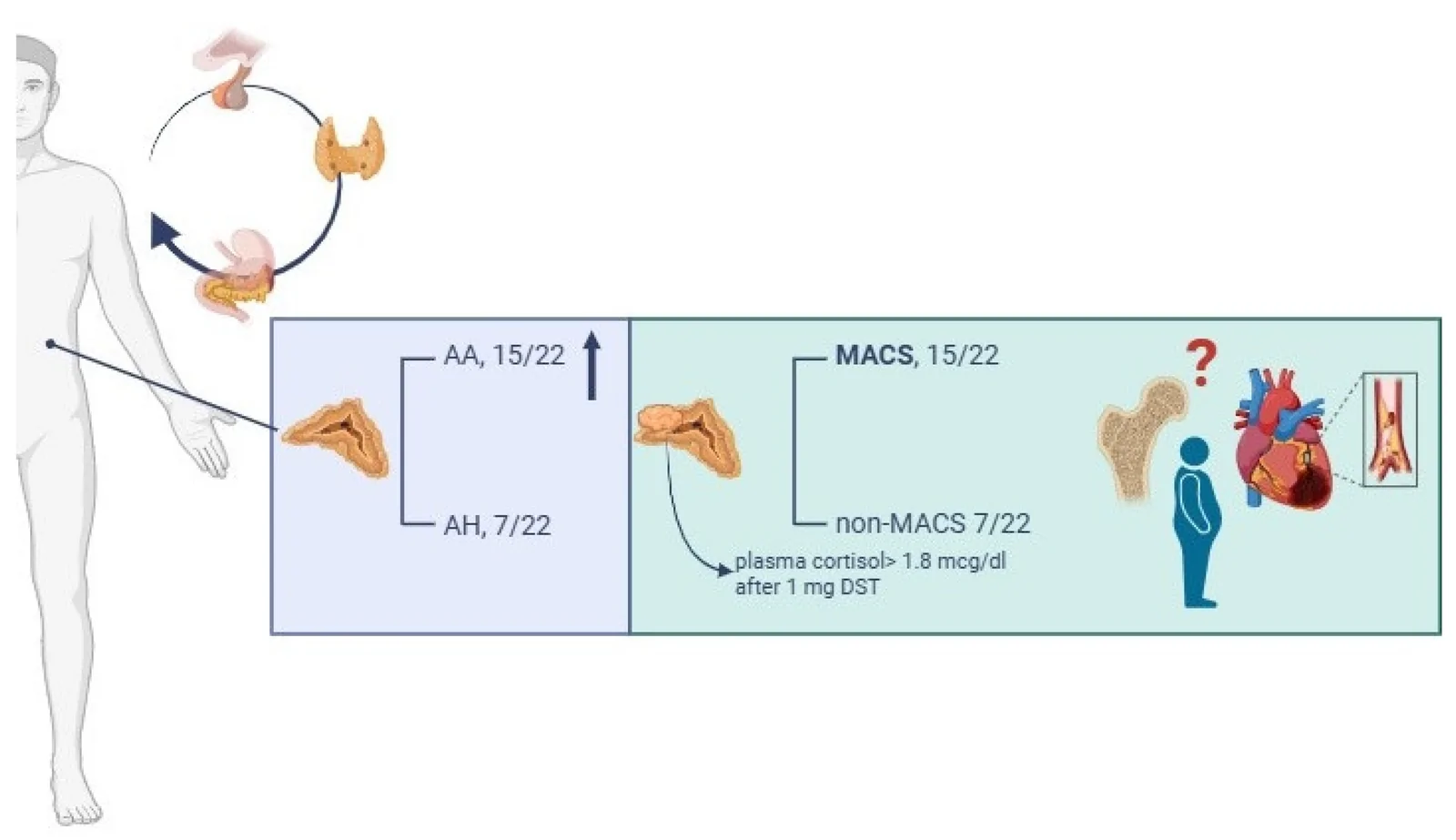
Distribution of adrenal involvement and mild autonomous cortisol secretion in the final analytic cohort of patients with multiple endocrine neoplasia type 1; Cardiometabolic and skeletal outcomes evaluated in the study are represented on the right side of the figure. Legend: 1 mg-DST: 1 mg dexamethasone suppression test; AA: adrenal adenoma; AH: adrenal hyperplasia; MACS: mild autonomous cortisol secretion.

In this retrospective monocentric cohort of 22 patients with MEN1 and radiological evidence of AA and/or AH, all subjects in the analytic cohort had an available 1 mg-DST, and MACS was identified in a relevant percentage of patients (68.2%). Adrenal lesions are acknowledged among MEN1 clinical manifestations, but published cohorts have primarily focused on their prevalence, morphology, growth, and malignant potential rather than on endocrine function [5,6,7,21] (Figure 1). Most of the current evidence concerning MACS derives from studies of patients with sporadic AI. Accordingly, current AI guidelines define MACS using post 1 mg-DST cortisol >1.8 µg/dL in the absence of overt Cushing syndrome, while recognizing substantial clinical heterogeneity of this condition [8]. MACS has consistently been associated with increased cardiometabolic risk and other adverse outcomes in patients with sporadic AI, thus its assessment may be clinically relevant in MEN1 patients [8,12]. Importantly, the prevalence of MACS in MEN1 is largely unexplored, and not systematically evaluated with the 1 mg-DST. In our series MACS prevalence appears higher than in sporadic AI cohorts, but this comparison should be interpreted cautiously because of substantial differences in study populations, referral patterns, imaging practices, and hormonal assessment [8,12]. Although the prevalence of pHPT in our cohort (90.9%) was consistent with the high penetrance reported in MEN1, the presence of dpNETs in all included patients may be higher than generally reported. This finding may at least partly reflect referral and detection bias, as our institution is an ENETS Center of Excellence and therefore follows a selected population with NET who undergo regular abdominal imaging as part of dpNET surveillance. Consequently, otherwise asymptomatic adrenal lesions may have been more frequently detected, and the composition of our cohort may limit the generalizability of these findings to the overall MEN1 population. Cardiometabolic comorbidities are central to the MACS framework, but in MEN1 they coexist with a multisystem endocrine syndrome that includes pHPT, which itself may be associated with cardiovascular and metabolic complications, and dpNET and PitNET that may contribute to metabolic morbidity through hormone-related syndromes [5,12,22,23,24]. In particular, the severity of pHPT may independently affect BMD and fracture risk, while functioning dpNETs and PitNETs may influence glucose metabolism, body composition, lipid profile, and cardiovascular risk. These overlapping endocrine manifestations complicate the attribution of cardiometabolic and skeletal abnormalities specifically to MACS. Moreover, treatment with SSA, beyond biliary stones and its acknowledged complications, has been associated with type 2 diabetes onset and worsening glycemic control, potentially confounding the attribution of cardiometabolic abnormalities to MACS [25,26,27]. Hyperprolactinemia, including its association with hypogonadism, may also contribute to adverse cardiometabolic outcomes, while emerging evidence also links hypoprolactinemia to impaired glucose and lipid metabolism and increased cardiometabolic risk [3,28]. pHPT, the earliest and most prevalent manifestation of MEN1, is a well-established determinant of reduced BMD and increased fracture risk, thus bone health impairment in MACS warrants careful interpretation [5]. In addition, studies in sporadic AA cohorts have revealed an increased prevalence of vertebral fractures and skeletal fragility among patients with MACS or AA outside MEN1 suggesting that fracture burden may be increased in patients with AA [29]. DHEA-S has been proposed as an indirect marker of chronic ACTH suppression because its circulating concentration is more stable than that of ACTH or unconjugated DHEA [10]. In patients with sporadic AI several studies have suggested that reduced DHEA-S concentrations, alone or in combination with ACTH, may aid in identifying MACS [10,11,30]. Low DHEA-S has been investigated in sporadic AI cohorts, including studies evaluating age- and sex-adjusted DHEA-S ratios or absolute DHEA-S thresholds [10,11,30]. Nevertheless, DHEA-S should not be considered diagnostic of MACS in MEN1-associated AA, and cut-offs proposed in non-MEN1 populations should not be assumed to apply to this specific hereditary setting. To our knowledge, the diagnostic value of DHEA-S has not previously been explored in MEN1-associated adrenal lesions. In the present cohort, DHEA-S suppression was numerically more frequent among patients with MACS, but the between-group difference was not statistically significant. DHEA-S concentrations depend on age, sex, assay characteristics, and the applicable reference interval, while the small sample precluded meaningful adjustment for these factors [31]. DHEA-S should therefore be considered a complementary biomarker rather than a substitute for the 1 mg-DST. Larger prospective studies are needed to determine whether DHEA-S provides additional diagnostic value in this specific clinical setting. Cardiometabolic comorbidities including hypertension, abnormalities of glucose metabolism, lipid disorders, and overweight or obesity were common in the present cohort, with no statistically significant differences observed between patients with and without MACS. In sporadic adrenal incidentaloma cohorts, MACS has been associated with increased cardiometabolic morbidity, particularly hypertension and disturbances of glucose metabolism, although the strength and consistency of these associations vary across studies [12,32,33,34]. Conversely, some studies have also reported similar cardiometabolic profiles in patients with autonomous cortisol secretion and apparently nonfunctioning adrenal adenomas, highlighting the difficulty of disentangling the metabolic effects of mild cortisol excess from those of aging, obesity, and other cardiovascular risk factors [35]. The present results neither support nor refute an association between MACS and cardiometabolic comorbidity in MEN1. The comparisons were based on small groups and were not adjusted for age, sex, BMI, diabetes, MEN1-related manifestations, or treatment. In addition, most patients were receiving SSA therapy, and all had dpNET. These characteristics complicate attribution of the observed metabolic burden specifically to MACS. Nevertheless, the lack of statistically significant differences should be considered in the context of a disease shaped by multiple, overlapping patient-, disease-, and treatment-related factors. Bone health impairment including low bone mass, osteoporosis, and vertebral fractures, were common in our cohort. Previous studies in sporadic adrenal adenoma have reported an increased fracture burden, while more recent studies have associated MACS with prevalent or incident vertebral fractures [29,36,37]. In the present cohort, neither osteoporosis nor vertebral fractures were more frequent among patients with MACS. The lower femoral-neck T/Z-score observed in the non-MACS group was an isolated finding and should not be interpreted as evidence of a protective effect of cortisol autonomy on bone health. Bone impairment in MEN1 is multifactorial, and pHPT, which affected most of our cohort, is likely to have been a major determinant. In addition, age, sex, gonadal status, pHPT, the functional status of dpNET and PitNET, and disease-specific treatments may all have influenced cardiometabolic and bone health outcomes; however, the relatively limited sample size precluded multivariable adjustment for these potential confounders, preventing the independent contribution of MACS from being determined. Accordingly, the present findings do not support a direct association between MACS and bone impairment in patients with MEN1, although the association cannot be excluded. To our knowledge, this is the first study specifically designed to investigate the prevalence of MACS in patients with MEN1 and adrenal lesions, thereby addressing an important gap in the current literature. All patients included in the final analytic cohort underwent a 1 mg-DST, ensuring complete ascertainment of the primary endpoint and minimizing the risk of misclassification. The availability of DHEA-S measurements in all patients also enabled an exploratory evaluation of this complementary biomarker in a clinical setting in which it has not previously been investigated. Furthermore, the integration of radiological, hormonal, cardiometabolic, and skeletal data provided a comprehensive clinical characterization of the cohort. Finally, the single-center design ensured relatively homogeneous diagnostic work-up and clinical management. On the contrary, the retrospective, single-center design and the relatively limited sample size, reflecting the rarity of MEN1, restrict the precision of the estimates and the generalizability of findings (e.g., BMI, impaired fasting glucose and femoral neck T/Z score). Furthermore, fasting insulin levels and specific indices of insulin resistance were not systematically available. The relatively limited number of MACS and non-MACS patients prevented robust multivariable analyses and reduced the ability to detect statistically significant differences between groups. Furthermore, pHPT high prevalence and severity, functioning dpNETs and PitNETs, and disease-specific treatments, including SSA therapy, may have independently influenced cardiometabolic and skeletal outcomes. Because of the small sample size and the heterogeneity of MEN1-related manifestations and treatments, adjustment for these potential confounders was not statistically feasible. Therefore, the comparisons between MACS and non-MACS patients cannot isolate the specific contribution of MACS. Consequently, the observed associations should be considered exploratory and hypothesis-generating rather than indicative of a causal relationship and should be interpreted accordingly.

## 5. Conclusions

The present study contributes novel evidence on the frequency of MACS in MEN1-associated adrenal lesions and supports the evaluation of cortisol secretion in selected MEN1 patients. MACS was frequently identified in our cohort, suggesting that biochemical evaluation of cortisol secretion should be included in MEN1 patients with adrenal involvement. DHEA-S suppression should currently be regarded as an exploratory complementary biomarker and not as a substitute for the 1 mg-DST. Cardiometabolic and skeletal impairment were common across the cohort; however, the small sample size and the potential confounding effects of other MEN1-related manifestations and treatments precluded assessment of the specific contribution of MACS to these outcomes. The present findings provide new evidence in a clinical setting for which functional adrenal data remain scarce, although no causal relationship between MACS and cardiometabolic or bone health can be assumed. Larger multicenter prospective studies incorporating standardized hormonal and imaging protocols, longitudinal evaluation of cardiometabolic and bone health outcomes, are needed to define the prevalence, natural history, and clinical significance of MACS in MEN1 and to establish whether a MEN1-specific approach to the diagnosis and management of MACS may translate into improved long-term clinical outcomes.

## Figures and Tables

**Table 1:** Baseline characteristics of patients with adrenal involvement.

Category	*n*	%
Total MEN1 population	101	100
Sex	59 F/42 M	
Patients with adrenal involvement	38	37.6%
Final study population	22	57.9%
Sex	10 F/12 M	
Mean age, years	57.3 (36–80)	
pHPT	20	90.9%
dpNET	22	100%
PitNET	13	59%
Bilateral adrenal involvement	10	45.5%
Left sided adrenal involvement	9	40.9%
Right sided adrenal involvement	3	13.6%
AA	15	68.2%
AH	7	31.8%
1 mg-DST	22	100%
MACS	15	68.2%
Non-MACS	7	31.8%
DHEA-S suppression	12	54.5%
BMI ≥ 25 kg/m^2^	16	72.7%
Arterial hypertension	11	50%
Diabetes	10	45.5%
Impaired fasting glucose	3	13.6%
Dyslipidemia	14	63.6
Metabolic syndrome	5	22.7%
SSA therapy	20	90.9%
Osteoporosis	12	57.1% *
Osteopenia	6	28.6% *
Fracture	7	33.3% *

Legend: AA: adrenal adenoma; AH: adrenal hyperplasia; BMI: body mass index; dpNET: duodenopancreatic neuroendocrine tumor; 1 mg-DST: 1 mg dexamethasone suppression test; F: female; M: male; MACS: mild autonomous cortisol secretion; MEN1: multiple endocrine neoplasia type 1; pHPT: primary hyperparathyroidism; PitNET: pituitary neuroendocrine tumor; SSA: somatostatin analogs. * Marked percentages were calculated based on a total of 21 patients.

**Table 2:** Continuous variables in the study population.

Category	*n*	Mean (Range)
Morning ACTH (pg/mL)	22	25.4 (10–95.6)
Morning cortisol (µg/dL)	22	17.1 (8.2–36.6)
24h-UFC (µg/24 h)	21	198.1 (21–477)
Post 1 mg-DST cortisol (µg/dL)	22	2.1 (0.9–3.9)
DHEA-S (µg/dL)	22	61.3 (1.95–232)
BMI (kg/m^2^)	22	27.9 (21.7–48.8)
Fasting glucose (mg/dL)	22	112.5 (82–219)
HbA1c, %	22	5.9 (4.3–9.1)
Total cholesterol (mg/dL)	22	158.2 (97–222)
HDL cholesterol (mg/dL)	22	57.9 (35–100)
LDL cholesterol (mg/dL)	22	82.5 (43–129)
Triglycerides (mg/dL)	22	96.3 (32–138)
Lumbar spine T-score/Z-score (SD)	21	−0.9 (−3.2–1.2)
Femoral neck T-score/Z-score (SD)	21	−1.3 (−3.3–2.9)

Legend: ACTH: adrenocorticotropic hormone; BMI: body mass index; DHEA-S: dehydroepiandrosterone sulfate; 1 mg-DST: 1 mg dexamethasone suppression test; HbA1c: glycated hemoglobin; HDL: high-density lipoprotein; LDL: low-density lipoprotein; SD: standard deviation; 24h-UFC: 24 h urinary free cortisol.

**Table 3:** Comparison between MACS and non-MACS patients.

Variable	MACS (*N*= 15)	Non-MACS (*N* = 7)	*p*-Value
Age, years	55.8 (36–75)	60.6 (41–80)	0.480
Female sex, *n/N* (%)	4/15 (26.7)	6/7 (85.7)	0.020
Bilateral adrenal involvement, *n/N* (%)	6/15 (40)	4/7 (57.1)	0.652
Morning ACTH, pg/mL	17.8 (10–28.2)	41.7 (16–95.6)	0.008
Morning cortisol, µg/dL	16 (8.2–30.3)	19.4 (10.4–36.6)	0.259
24h-UFC, µg/24 h	212.4 (33–477); *n* = 14	169.6 (21–307)	0.526
Post 1 mg-DST cortisol, µg/dL	2.5 (1.8–3.9)	1.2 (0.9–1.8)	<0.001
DHEA-S, µg/dL	45 (2–226.1)	96.3 (11–232.6)	0.139
Suppressed DHEA-S, *n/N* (%)	10/15 (66.7)	2/7 (28.6)	0.172
BMI, kg/m^2^	27.8 (22.6–31.6)	28.2 (21.7–48.8)	0.147
BMI ≥ 25 kg/m^2^ *n/N* (%)	12/15 (80)	4/7 (57.1%)	0.334
BMI ≥ 30 kg/m^2^ *n/N* (%)	2/15 (13.3)	1/7 (14.3%)	1.000
Arterial hypertension, *n/N* (%)	6/15 (40)	5/7 (71.4)	0.361
Impaired fasting glucose *n/N* (%)	2/15 (13.3)	1/7 (14.3)	1.000
Diabetes therapy, *n/N* (%)	8/15 (53.3)	2/7 (28.6)	0.381
Fasting glucose, mg/dL	117.1 (86–219)	102.6 (82–136)	0.307
HbA1c, %	6.0 (4.3–9.1)	5.6 (4.3–7.1)	0.502
Dyslipidemia *n/N* (%)	10/15 (66.7)	4/7 (57.1)	1.000
Lipid-lowering therapy, *n/N* (%)	9/15 (60)	3/7 (42.9)	0.652
Total cholesterol, mg/dL	156.5 (97–222)	162.0 (114–197)	0.724
LDL cholesterol, mg/dL	85.7 (47–129)	75.7 (43–95)	0.378
Triglycerides, mg/dL	96.7 (32–138)	95.3 (61–136)	0.944
HDL cholesterol, mg/dL	53.2 (35–82)	68.0 (42–100)	0.057
Metabolic syndrome, *n/N* (%)	4/15 (26.7)	1/7 (14.3)	1.000
SSA therapy, *n/N* (%)	13/15 (86.7)	7/7 (100)	1.000
Osteoporosis, *n/N* (%)	7/14 (50)	5/7 (71.4)	0.642
Osteopenia, *n/N* (%)	5/14 (35.7)	1/7 (14.3)	0.613
Lumbar spine T-score/Z-score, SD	−0.7 (−3.2–1.2); *n* = 14	−1.4 (−2.5–0.6)	0.247
Femoral neck T-score/Z-score, SD	−0.7 (−2.5–2.9); *n* = 14	−2.3 (−3.3 to −0.5)	0.023
Fracture, *n/N* (%)	5/14 (35.7)	2/7 (28.6)	1.000

Legend. ACTH: adrenocorticotropic hormone; BMI: body mass index; DHEA-S: dehydroepiandrosterone sulfate; 1 mg-DST: 1 mg dexamethasone suppression test; HbA1c: glycated hemoglobin; HDL: high-density lipoprotein; LDL: low-density lipoprotein; MACS: mild autonomous cortisol secretion; SD: standard deviation; SSA: somatostatin analog; 24h-UFC: 24 h urinary free cortisol.

## Data Availability

The data presented in this study are not publicly available due to ethical and privacy restrictions, as they contain sensitive clinical information. Data supporting the findings of this study are available from the corresponding author upon reasonable request and with permission of the local ethics committee.

## Abbreviations

The following abbreviations are used in this manuscript:

1 mg-DST	1 mg dexamethasone suppression test
24h-UFC	24 h urinary free cortisol
AA	adrenal adenoma
ACTH	adrenocorticotropic hormone
AH	adrenal hyperplasia
AHA/NHLBI	American Heart Association/National Heart, Lung and Blood Institute
AI	adrenal incidentaloma
BMD	bone mineral density
BMI	body mass index
DHEA-S	dehydroepiandrosterone sulfate
dpNET	duodenopancreatic neuroendocrine tumor
DXA	dual-energy X-ray absorptiometry
EASO	European Association for the Study of Obesity
ENETS	European Neuroendocrine Tumor Society
HbA1c	glycated hemoglobin
HDL	high-density lipoprotein
LDL	low-density lipoprotein
MACS	mild autonomous cortisol secretion
MEN1	multiple endocrine neoplasia type 1
pHPT	primary hyperparathyroidism
PitNET	pituitary neuroendocrine tumor
SD	standard deviation
SSA	somatostatin analog
